# Thoracic Aortic Calcification and Pre-Clinical Hypertension by New 2017 ACC/AHA Hypertension Guidelines

**DOI:** 10.3390/diagnostics11061027

**Published:** 2021-06-03

**Authors:** Ya-Ting Jan, Pei-Shan Tsai, Chris T. Longenecker, Dao-Chen Lin, Chun-Ho Yun, Kuo-Tzu Sung, Chuan-Chuan Liu, Jen-Yuan Kuo, Chung-Lieh Hung, Tung-Hsin Wu, Jiun-Lu Lin, Charles Jia-Yin Hou, Cheng-Ting Tsai, Chen-Yen Chien, Aaron So

**Affiliations:** 1 Department of Biomedical Imaging and Radiological Sciences, National Yang Ming Chiao Tung University, Taipei 112, Taiwan; gracilis0328@gmail.com (Y.-T.J.); purifying@gmail.com (P.-S.T.); 8905012@gmail.com (K.-T.S.); 2 Department of Radiology, MacKay Memorial Hospital, Taipei 104, Taiwan; med202657@gmail.com; 3 Department of Medicine, Mackay Medical College, New Taipei City 252, Taiwan; jykuo5813@gmail.com (J.-Y.K.); jotaro3791@gmail.com (C.-L.H.); jiayin@mmh.org.tw (C.J.-Y.H.); chengtingtsai@gmail.com (C.-T.T.); 4 Mackay Junior College of Medicine, Nursing and Management, New Taipei City 112, Taiwan; 5 University Hospitals Cleveland Medical Center, Cleveland, OH 44106, USA; longenc2@gmail.com; 6 Division of Endocrine and metabolism, Department of Medicine, Taipei Veterans General Hospital, Taipei 112, Taiwan; dclin@vghtpe.gov.tw; 7 Department of Radiology, Taipei Veterans General Hospital, Taipei 112, Taiwan; 8 School of Medicine, National Yang Ming Chiao Tung University, Taipei 112, Taiwan; 9 Division of Cardiology, Department of Internal Medicine, MacKay Memorial Hospital, Taipei 104, Taiwan; 10 Graduate Institute of Health Care Organization Administration, College of Public Health National Taiwan University, Taipei 100, Taiwan; carrie@ms1.mmh.org.tw; 11 Health Evaluation Center, MacKay Memorial Hospital, Taipei 104, Taiwan; 12 Department of Medical Technology, Yuanpei University of Science and Technology, Hsin-Chu City 306, Taiwan; 13 Division of Endocrinology and Metabolism, Department of Internal Medicine, MacKay Memorial Hospital, Taipei 104, Taiwan; jiunlulin@gmail.com; 14 Division of Cardiovascular Surgery, Department of Surgery, MacKay Memorial Hospital, Taipei 104, Taiwan; 15 Imaging Program, Lawson Health Research Institute, London, ON N6C 2R5, Canada; aso@robarts.ca; 16 Department of Medical Biophysics, University of Western Ontario, London, ON N6A 3K7, Canada

**Keywords:** hypertension, multi-detector computed tomography, thoracic aorta calcification

## Abstract

The recently revised 2017 American College of Cardiology/American Heart Association (ACC/AHA) hypertension (HTN) guidelines employ a lower blood pressure threshold to define HTN, aiming for earlier prevention of HTN-related cardiovascular diseases (CVD). Thoracic aortic calcification (TAC), a new surrogate marker of aging and aortic medial layer degeneration, and different stages of HTN, according to the 2017 ACC/AHA HTN guidelines, remain unknown. We classified 3022 consecutive asymptomatic individuals enrolled into four HTN categories using the revised 2017 ACC/AHA guidelines: normal blood pressure (NBP), elevated blood pressure (EBP), and stage 1 (S1) and stage 2 (S2) HTN. The coronary artery calcification score and TAC metrics (total Agaston TAC score, total plaque volume (mm^3^), and mean density (Hounsfield units, HU)) were measured using multi-detector computed tomography. Compared to NBP, a graded and significant increase in the TAC metrics was observed starting from EBP and S1 and S2 HTN, using the new 2017 ACC/AHA guidelines (NBP as reference; all trends: *p* < 0.001). These differences remained consistent after being fully adjusted. Older age (>50 years), S1 and S2 HTN, prevalent diabetes, and chronic kidney disease (<60 mL/min/1.73 m^2^) are all independently contributing factors to higher TAC risk using multivariate stepwise logistic regressions (all *p* ≤ 0.001). The optimal cutoff values of systolic blood pressure, diastolic blood pressure, and pulse pressure were 121, 74, and 45 mmHg, respectively, for the presence of TAC after excluding subjects with known CVD and ongoing HTN medication treatment. Our data showed that the presence of TAC starts at a stage of elevated blood pressure not categorized as HTN from the updated 2017 ACC/AHA hypertension guidelines.

## 1. Introduction

Hypertension (HTN) is one of the most important cardiovascular risk factors, and accounts for approximately half of all cardiovascular disease (CVD) events worldwide [1]. In 2017, the American College of Cardiology (ACC) and the American Heart Association (AHA) jointly revised the guidelines for the detection, evaluation, and prevention of hypertension (hereafter referred to as “the 2017 ACC/AHA HTN guidelines” or simply “the revised HTN guidelines”), in which the blood pressure threshold for the diagnosis of HTN has been lowered [2]. The revised HTN guidelines have led to swift changes in patient management, where more aggressive medical interventions are used in the pre-clinical stage to minimize the risk of future CVD events in hypertensive patients. Hypertension is a strong predictor of adverse cardiovascular events, and is a major contributing factor to atherosclerosis, central aortic stiffness, and heart failure (HF) [3].

Recent studies have shown that thoracic aorta calcification (TAC) is a potent marker of age-related pathological degeneration in the great arteries, and a predictor of major cardiovascular disorders and non-cardiovascular mortality [4,5,6,7,8]. Furthermore, several population-based epidemiological studies have revealed a high degree of association between hypertension and the TAC score, assessed using computed tomography (CT) the Caucasian populations [4,9,10,11]. However, it is unclear whether such strong association between hypertension and TAC holds true in the Asian population. 

The findings of previous studies have suggested that Asian populations are more susceptible to hypertension-related CVD at early stages of the disease relative to other ethnic groups [12,13]. In this regard, we speculated that the effect of blood pressure on great arterial damage, as manifested by aortic calcification in the pre-clinical stage and defined as elevated blood pressure not reaching the guideline-recommended HTN threshold [13,14], likely imitates HTN-related CVD in the Asian population.

As the correlation between TAC and the different levels of hypertension classified in the revised HTN guidelines has not been fully established, the objective of this study was to investigate the correlations between the TAC burden assessed by multi-detector computed tomography (MDCT) (such as volume and density of calcification), blood pressure metrics (including systolic blood pressure, diastolic blood pressure, and pulse pressure (SBP, DBP, and PP, respectively)), and other cardiovascular risk factors in a large cohort of Asian patients with hypertension, according to the 2017 ACC/AHA guidelines [2].

## 2. Materials and Methods

### 2.1. Study Population

From January 2005 to December 2012, a total of 3120 consecutive subjects underwent health surveys and non-contrast-enhanced MDCT scan for assessment of the coronary artery calcium score in the health promotion and evaluation center, Mackay Memorial Hospital, Taipei, Taiwan. Of these patients, 3022 (2161 men and 861 women, mean age = 49.4 ± 9.5 years) who had complete clinical and MDCT data were enrolled in this study. The clinical and CT data were analyzed retrospectively and de-identified prior to author access and analysis. The study was approved by the Institutional Research Ethics Board (IRB) of MacKay Memorial Hospital in Taipei, Taiwan (17MMHIS082e), and the requirement for written patient consent was waived by the IRB.

The enrolled subjects were divided into four study groups according to their blood pressure measurements and the 2017 ACC/AHA HTN guidelines: (1) normal blood pressure (NBP), defined as SBP less than 120 mmHg and DBP less than 80 mmHg; (2) pre-clinical stage of HTN as elevated blood pressure (EBP), defined as SBP between 120 and 129 mmHg and DBP less than 80 mmHg; (3) stage 1 hypertension (S1 HTN), defined as SBP between 130 and 139 mmHg or DBP between 80 and 89 mmHg; and (4) stage 2 hypertension (S2 HTN), defined as an SBP greater than or equal to 140 mmHg or DBP greater than or equal to 90 mmHg, or with a known history of hypertension. Hypertension medication use referred to the use of renin–angiotensin system (RAS) inhibitors, calcium channel blockers, alpha- or beta-blockers, and diuretics prescribed by physicians aiming for treatment of hypertension.

In comparison, the four categories of hypertension defined in the 2018 ESH/ESC guidelines for the management of arterial hypertension [14] were as follows: (1) NBP, defined as SBP less than 130 mmHg and DBP less than 85 mmHg; (2) high normal blood pressure (HNBP), defined as SBP between 130 and 139 mmHg and/or DBP between 85 and 89 mmHg; (3) S1 HTN, defined as SBP between 140–159 mmHg and/or DBP between 90–99 mmHg; and (4) S2 HTN and above, defined as SBP ≥ 160 mmHg and/or DBP ≥ 100 mmHg. Among those diagnosed with HTN, 237 subjects (80%) had ongoing HTN medication control at the time of study enrollment. The study setting and design had been previously published [15].

### 2.2. Anthropometric Measures and Biochemical Data

The baseline characteristics and biochemical data, including age, sex, body mass index (BMI), body fat composition, waist–hip ratio (WHR), and lipid profiles, including total cholesterol, triglycerides, LDL cholesterol, HDL cholesterol, estimated glomerular filtration rate (eGFR), high-sensitivity C-reactive protein (hs-CRP), and homocysteine level were collected. A thorough review of the subject’s health history, including the use of dyslipidemia medication, known CVD (such as stroke or coronary artery disease), diabetes, and smoking habits, was also conducted via detailed questionnaires. Resting blood pressure was measured using a standardized sphygmomanometer by the medical staff, who were blinded to the other test results. 

### 2.3. Semi-Automatic Quantification of Three-Dimensional (3D) Thoracic Aortic Calcification

**Figure 1:**
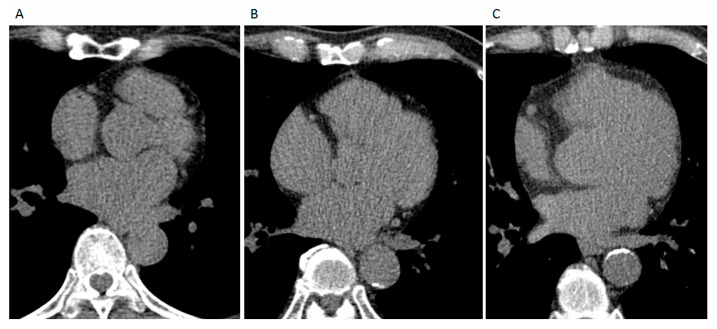
Non-enhanced, ECG-gated cardiac MDCT images revealed (**A**) no calcification of thoracic aorta in a patient with normal blood pressure (NBP), (**B**) mild calcification in a patient with elevated blood pressure (EBP), and (**C**) moderate to severe calcification in a patient with severe hypertension (S2 HTN), according to the 2017 ACC/AHA HTN guidelines.

Cardiac CT was performed using a 16-slice MDCT scanner (Sensation 16, Siemens Medical Solutions, Forchheim, Germany) with 16 × 0.75 mm collimation, a 420 ms gantry rotation period, and 120 kV tube voltage. Transaxial images were acquired from the point above the level of the tracheal bifurcation to the point below the base of the heart in a single breath-hold using prospective electrocardiogram triggering, with the center of the acquisition window set at 70% of the R–R interval. Transaxial images were reconstructed from the scan data with a standard image reconstruction kernel at 3 mm slice thickness without overlapping and with a 25 cm display field of view. All image analyses were performed on a dedicated workstation (Aquarius 3D Workstation, TeraRecon, San Mateo, CA, United States). The Agatston score, volume score (mm^3^), and density score (HU) were measured to quantify arterial calcification and for coronary artery calcification (CAC) (using the Agatston score). TAC in the ascending or descending aorta was defined as a region covering at least four contiguous pixels with an average intensity greater than 130 Hounsfield units (HU) [16]. Subsequently, the TAC score was calculated by multiplying the area of each lesion by a weighting factor associated with the lesion [16]. The weighting factor was assigned in the following manner: 1 = lesion with a density between 130 and 199 HU; 2 = lesion with a density between 200 and 299 HU; 3 = lesion with a density between 300 to 399 HU; and 4 = lesion with a density greater than 400 HU. The total calcium score was calculated by summing the individual lesion scores. In this dedicated workstation, in addition to the TAC score, the TAC volume (the total volume of all lesions measured in mm^3^) and TAC mean density (mean density in HU of all selected calcifications) were also demonstrated (Figure 1).

### 2.4. Statistical Analysis

Continuous variables were expressed as mean ± standard deviation (SD), and categorical data were presented as numbers and percentages. All statistical analyses were performed using the IBM SPSS Statistics software version 22 (IBM Corp. Armonk, NY, USA). The between-group differences were assessed using analysis of variance and the chi-squared test. The correlations between the TAC burden and BP components (as continuous variables) were assessed by calculating the Pearson product–moment correlation coefficient. The chi-squared test and logistic regression were used to establish the risk of TAC (odds ratio (OR) and 95% confidence interval (CI)) associated with the BP components. A multivariate, forward, stepwise logistic regression analysis was used to explore the association between BP and TAC severity. A general linear model was used to analyze the effects of multiple variables and to obtain the adjusted estimation of various TAC measures (presented as mean ± standard error) without assuming that the response is normally distributed, according to the 2017 ACC/AHA HTN guidelines. A sensitivity analysis was conducted by excluding the subjects with an ongoing use of HTN medications (*n* = 237) and known CVD (*n* = 133) from analysis.

To examine the extent of blood pressure components that may affect the presence of aortic calcification, the optimal cutoffs of SBP, DBP, and PP (as independent variables) in the presence of TAC as an outcome measure were examined. Analysis was conducted by using the area under the receiver operating characteristic (ROC) curves to define the optimal cutoffs from various BP components (including SBP, DBP, and PP) to identify the presence of TAC by the maximal Youden index (largest summation of sensitivity and specificity). A sensitivity analysis was also conducted by excluding those with an ongoing HTN treatment and those with known CVD.

## 3. Results

### 3.1. Baseline Demographics

The detailed clinical characteristics and baseline biochemical data are summarized in Table 1. There were 1204, 591, 383, and 844 study subjects classified into the NBP, EBP, S1 HTN, and S2 HTN categories, respectively, according to the 2017 ACC/AHA definition of hypertension [2]. On average, the subjects in the S2 HTN group were older, had a higher BMI, a higher WHR, and greater body fat composition than those in the other three groups. The subjects in the EBP, S1 HTN, and S2 HTN groups had higher levels of HbA1c, total cholesterol, triglyceride, LDL cholesterol, and uric acid, but a lower HDL cholesterol levels compared to the NBP group. However, hs-CRP and homocysteine levels were only significantly higher in the S2 HTN group than in the other HTN groups. A clinical history, including dyslipidemia, CVD, stroke, and diabetes, was more likely to be present in the S2 HTN group. 

### 3.2. Coronary and Thoracic Aortic Calcification across Four Categories by ACC/AHA Hypertension Guidelines

The prevalence of CAC, TAC, and carotid plaque were 19.7%, 7.6%, and 4.4%, respectively, in the NBP group; 29.6%, 13.2%, and 5.6%, respectively, in the EBP group; 35.5%, 21.1%, and 7.3%, respectively, in the S1 HTN group; and 51.1%, 30.2%, 13.9%, respectively, in the S2 HTN group. Positive and significant correlations between the CAC score and TAC score, volume, and mean density were observed (*r* = 0.28, 0.30, and 0.33, respectively; all *p* < 0.001). The individuals with TAC were also more likely to have CAC (OR = 7.03 (95% CI: 5.69–8.68), *p* < 0.001), which remained significant after full adjustment (adjusted OR = 3.47 (95% CI: 2.70–4.44), *p* < 0.001). The TAC score, volume, and mean density showed a graded and significant increase across different HTN stages/grades defined by the 2017 ACC/AHA HTN and the 2018 ESC/ESH HTN guidelines, even after full adjustment (Table 2). However, such differences in the CAC score across different HTN stages/grades were no longer significant after adjustment (Table 2). These findings were broadly consistent with the elimination of those with known CVD and subjects with ongoing use of HTN medications (final *n* = 2784) in our sensitivity analysis (Supplemental Table S1).

### 3.3. Associations of Blood Pressure Components with Coronary and Thoracic Aortic Calcification

**Figure 2:**
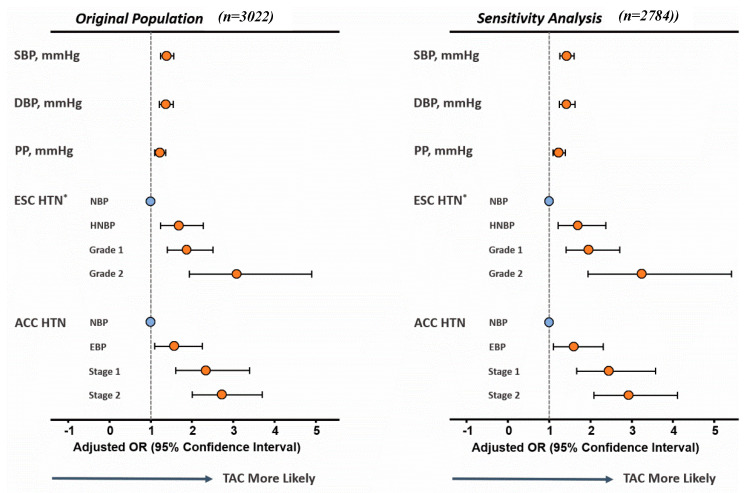
Probability of the presence of TAC, predicted by each blood pressure component (SBP, DBP and PP), and HTN stages defined in the 2017 ACC/AHA HTN guidelines in original cohort (left), compared with results obtained from the sensitivity analysis (right), which excluded the subjects with known CVD and ongoing HTN medication use.

Each higher blood pressure component and more advanced HTN independently showed a higher TAC probability (Figure 2), starting from EBP and HNBP stage/grade, respectively. A multivariate logistic regression analysis revealed that SBP and PP were significant predictors of TAC. Using the TAC score, volume, and mean density as dependent outcome measures (continuous variables), per 1 standardized increment of SBP (adjusted coefficient = 52.1, 41.3, and 11.5, respectively; 95% CI: 31.1–73.0, 24.2–58.5, and 8.10–14.9, respectively; all *p* < 0.001), and PP (adjusted coefficient = 57.1, 9.00, and 11.4, respectively; 95% CI = 37.0–77.1, 31.2–64.0, and 8.1–14.7, respectively; all *p* < 0.001) were independently associated with a higher TAC burden by three different TAC measures in multivariate regression analysis, although only the TAC mean density was significantly associated with DBP in the adjusted model (adjusted coefficient = 4.61; 95% CI: 1.2–8.0, *p* = 0.008). Older age (>50 years), higher blood pressure (including ACC/AHA-defined stage 1 and stage 2 HTN), prevalent diabetes, and chronic kidney disease (CKD) were likely the main determinants of TAC development. Male sex, age more than 50 years, BMI > 25 kg/m^2^, higher blood pressure (including ACC/AHA-defined S1 and S2 HTN), and diabetes were statistically independent predictors of CAC (Table 3). The presence of TAC showed a low sensitivity (25%), albeit with a high specificity (91%) for identifying early stages (1 and 2) HTN. The sensitivity analysis yielded similar results (Supplemental Table S2).

**Figure 3:**
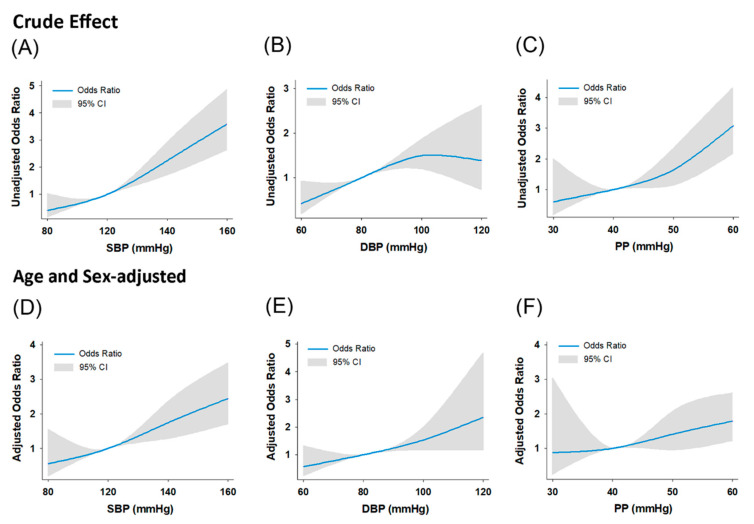
Probability of the presence of TAC predicted by the systolic blood pressure (SBP), diastolic blood pressure (DBP), and pulse pressure (PP), in subjects without known CVD or ongoing use of HTN medications (*n* = 2784) (**A**–**C**), as well as after age and sex adjustment (**D**–**F**).

Overall, the ROC analysis of SBP revealed an area under the curve (AUC) of 0.65 and a cutoff value of 129 mmHg (54.52% sensitivity and 69.01% specificity). The ROC analysis of DBP revealed an AUC of 0.56 and a cutoff value of 71 mmHg (69.84% sensitivity and 40.51% specificity). The ROC analysis of PP revealed an AUC of 0.64 and a cutoff value of 44 mmHg (69.14% sensitivity and 51.60% specificity). The SBP, DBP, and PP were set at 128, 74, and 49 mmHg, respectively. The sensitivity analysis showed that the SBP, DBP, and PP at the optimal cutoff thresholds (121, 74, and 45 mmHg, respectively) had the largest area under the ROC curve for identifying the presence of TAC after eliminating the subjects with known CVD and ongoing use of HTN medications (remaining subject number for analysis was 2784). The relationship between each higher blood pressure component and the likelihood of TAC, after excluding known CVD and consumption of HTN medications, signified the importance of hypertension on the development of TAC at relatively lower blood pressure levels (Figure 3).

## 4. Discussion

Our findings suggest that advanced age, hypertension, and prevalent diabetes are strongly correlated with calcification in the aorta and coronary arteries. Individuals who were traditionally classified in the elevated blood pressure category (SBP range: 130–139 mmHg), but not classified as hypertensive, according to the updated ACC/AHA guidelines were associated with a higher TAC score than coronary calcification burden after detailed adjustment. Our findings on the presence of TAC as highly specific for early-stage HTN support the 2017 ACC/AHA HTN guidelines for lowering the target threshold of HTN management.

The results of the multi-ethnic study of atherosclerosis and the Heinz Nixdorf Recall study [10,11] collectively concluded that the clinical risks associated with CAC and TAC were similar. We confirmed a positive yet modest correlation between the CAC and TAC burden. Consistent with the study by Youssef et al., an advanced age (>50 years), early HTN defined by ACC/AHA guidelines, and diabetes medical history were the independent predictors for the presence of both CAC and TAC [10]. The shared pathophysiology of these conventional risk factors may induce a cascade of complex biological reactions, including elicited oxidative stress, vascular inflammation, endothelial dysfunction, and vascular smooth muscle cell trans-differentiation to osteoblast-like cells, resulting in the aortic calcification and greater central artery stiffness [17,18,19,20].

A previous study showed that a higher arterial afterload was independently correlated with TAC [21]. Other epidemiological studies have also reported the pathological link between aortic calcification and arterial stiffness [20,22,23,24,25,26,27], in which the arterial stiffness was mainly attributable to intima-media calcification rather than atheroma or atherosclerosis [28]. As aortic stiffness is known to be a marker of vascular aging and possibly serves as a driving factor for arterial hypertension in elderly individuals, it is possible that the relationship between hypertension and TAC is bidirectional, where hypertension may induce TAC and vice versa [29,30]. Conversely, central arterial calcification and its consequence of reduced aortic reservoir function and heightened arterial stiffness may pose a greater myocardial afterload and worsen ischemia; both symptoms are closely linked to the development of left ventricular hypertrophy (LVH) [20,31,32]. A retrospective study by Cho et al. [33] revealed that patients with both LVH and a higher TAC score had the worst clinical outcomes. As the complex interactions between the ventricular and arterial systems (known as ventricular–arterial coupling) may serve as a catalyst for the development of heart failure with a preserved ejection fraction (HFpEF), our findings that TAC accumulation may occur in the early stages of HTN may indicate an early therapeutic action based on a primary preventive standpoint [34].

Following the SPRINT trial [35,36], the blood pressure threshold used for the clinical diagnosis of HTN was lowered, which was immediately adopted by the 2017 ACC/AHA guidelines. Despite the observed concomitant increase in CAC, TAC, and carotid artery plaque across the HTN stages using ACC/AHA HTN guidelines in the present study, notably, we observed that the CAC differences across the ordered HTN stages were largely diminished in fully adjusted models. Instead, the presence of TAC, manifested especially by TAC mean density, showed a consistent and graded increase, starting from a blood pressure threshold (EBP stage according to the 2017 ACC/AHA HTN guidelines) traditionally not classified as HTN, according to the other criteria [14]. This finding probably revealed a closer pathological link between elevated afterload and large conduit artery, particularly the aorta. Our study is the first to report the relationship between TAC and the different HTN stages defined in the revised HTN guidelines. Although Damian et al. reported that the TAC volume was positively associated with blood pressure rather than TAC density [37], we speculated that this discrepancy was mainly due to the racial differences and disparities in baseline demographics. For example, our population presented a relatively healthy and pre-clinical population for cardiovascular risks. We observed a greater risk of TAC, which may begin in the early stages of HTN (starting from the EBP stage according to the 2017 ACC/AHA HTN guidelines, or from the NHBP grade according to the 2018 ESC/ESH guidelines), even in our fully adjusted model (NBP vs. EBP by ACC/AHA criteria, and NBP vs. NHBP by ESC/ESH criteria). Given the fact that the presence of TAC showed a high specificity for detecting subjects presenting elevated blood pressure prior to a well-defined HTN stage, our findings also signify and support heightened clinical CVD risks in a targeted population across the HTN spectrum in an Asian population.

This study had several limitations. First, our study was cross-sectional; therefore, we were unable to infer causality or determine the causal effects of blood pressure on aortic calcification. Additional longitudinal studies are needed to discern the temporal nature of these events. Second, information regarding the exact time point of hypertension onset and some clinical hypertension subgroups, such as those with masked hypertension or morning surge in blood pressure, were missing in our study samples. Hence, a prospective study may be needed to further elucidate the relationship between hypertension and TAC. Third, as greater TAC burden likely reflects increasing cardiovacular co-morbid conditions and positively correlates with higher blood pressure (especially systolic blood pressure) [10], the sensitivity of TAC as a screening marker for elevated blood pressure remains uncertain and limited. Fourth, as our findings were mainly drawn from an Asian (Chinese) population, subsequent investigations on non-Asian populations are warranted, in order to determine whether there are any racial differences in the association between hypertension and TAC.

In summary, we demonstrated a significant association between the TAC and HTN stages defined in the 2017 ACC/AHA guidelines in a large, asymptomatic Asian population. We also identified that advanced age (>50 years), HTN, diabetes, and CKD (eGFR <60 mL/min/1.73 m^2^) were all associated with the development of TAC. Furthermore, TAC may begin to develop in the pre-clinical stage of HTN, which highlights the importance of early diagnosis of and intervention for arterial hypertension in patients at high risk.

## Figures and Tables

**Table 1:** Clinical and baseline characteristics of patients with normal blood pressure (NBP), elevated blood pressure (EBP), and stage 1 (S1) and stage 2 (S2) hypertension (HTN).

	All Study Participants	NBP	EBP	S1 HTN	S2 HTN)	*p*-Value (Trend)
	(*n* = 3022)	(*n* = 1204)	(*n* = 591)	(*n* = 383)	(*n* = 844)	
Clinical feature						
Age (years)	49.4 ± 9.5	47.0 ± 8.6	48.4 ± 9.4 *	50.3 ± 9.8 *^a^	53.6 ± 9.6 *^bc^	<0.01
Male gender	2161 (71.5%)	779 (64.7%)	426 (72.1%)	296 (77.3%)	660 (78.2%)	<0.01
BMI (kg/m^2^)	24.69 ± 3.51	23.39 ± 2.97	24.67 ± 3.15 *	25.50 ± 3.62 *^a^	26.18 ± 3.72 *^bc^	<0.01
WHR	0.88 ± 0.07	0.86 ± 0.07	0.89 ± 0.06 *	0.90 ± 0.65 *^a^	0.91 ± 0.06 *^bc^	<0.01
Body fat (%)	26.08 ± 6.59	24.87 ± 6.04	25.98 ± 6.32 *	26.50 ± 6.90 *	27.74 ± 7.05 *^bc^	<0.01
Biochemistry data						
Hemoglobin A1c (%)	5.86 ± 0.88	5.66 ± 0.60	5.79 ± 0.77 *	5.98 ± 1.05 *^a^	6.13 ± 1.09 *^b^	<0.01
Total cholesterol (mg/dL)	201.96 ± 36.61	198.20 ± 35.62	203.93 ± 36.27 *	203.85 ± 34.93 *	205.09 ± 38.56 *^b^	<0.01
Triglyceride (mg/dL)	141.81 ± 116.10	122.51 ± 79.88	139.72 ± 81.96 *	153.97 ± 100.34 *	165.34 ± 169.71 *^b^	<0.01
LDL cholesterol (mg/dL)	130.48 ± 32.48	127.72 ± 32.31	133.54 ± 32.58 *	131.99 ± 31.75	131.61 ± 32.02*	NS
HDL cholesterol (mg/dL)	52.35 ± 14.09	54.68 ± 15.05	52.20 ± 13.93 *	50.88 ± 13.60 *^a^	49.78 ± 12.41 *	<0.01
Uric acid (mg/dL)	6.03 ± 1.44	5.73 ± 1.42	6.11 ± 1.41 *	6.15 ± 1.42 *	6.35 ± 1.44 *^b^	<0.01
eGFR (ml/min/1.73 m^2^)	82.81 ± 15.16	85.26 ± 15.72	83.73 ± 14.90	82.69 ± 15.74 *^a^	79.57 ± 16.49 *^bc^	<0.01
hs-CRP (mg/dL)	0.20 ± 0.41	0.16 ± 0.34	0.19 ± 0.35	0.20 ± 0.33	0.27 ± 0.56 *	<0.01
Homocysteine (µmol/L)	9.31 ± 4.04	8.86 ± 3.69	9.32 ± 3.93	10.00 ± 5.32	9.67 ± 3.96 *	<0.01
Medical History						
Dyslipidemia	158 (5.2%)	31 (2.5%)	22 (3.7%)	12 (3.1%)	93 (11.0%) *^bc^	<0.01
CVD	133 (4.4%)	26 (2.1%)	9 (1.5%)	9 (2.3 %)	89 (10.5%) *^bc^	<0.01
Stroke	13 (0.43%)	1 (0.08%)	1 (0.16%)	0 (0%)	11 (1.3%) *	<0.01
Diabetes	174 (5.7%)	21 (1.7%)	23 (3.9%) *	18 (4.7%) *	112 (13.3%) *^bc^	<0.01
Smoking	363 (12%)	153 (12.7%)	72 (12.2%)	40 (10.4%)	98 (11.6%)	NS

Data presented as mean ± SD or numbers (%). NBP: normal blood pressure; EBP: elevated blood pressure; S1 HTN: stage 1 hypertension; S2 HTN: stage 2 hypertension; BMI: body mass index; WHR: waist-to-hip ratio; eGFR: estimated Glomerular filtration rate; hs-CRP: high-sensitivity C-reactive protein; CAC: coronary artery calcification; CVD: cardiovascular disease; NS: non-significant; TAC: thoracic artery calcification. * *p* < 0.01 compared to NBP, ^a^ *p* < 0.01 for comparison between S1 HTN and EBP, ^b^ *p* < 0.01 for comparison between S2 HTN and EBP, ^c^ *p* < 0.01 for comparison between S2 HTN and S1 HTN.

**Table 2:** Adjusted estimates of thoracic aortic calcification (TAC) burden from different measures across different stages/grades of HTN, using different clinical guidelines.

ACC/AHA 2017 HTN Guideline [2]						
(Original Population, *n* = 3022)						
BP Category (mmHg)	SBP	DBP	TAC Score	TAC Volume (mm^3^)	TAC Density (HU)	CAC Score
Normal (NBP)	<120	and <80	35.3 ± 13.6	32.4 ± 11.1	35.2 ± 2.20	36.0 ± 5.4
Elevated (EBP)	120–129	and <80	63.4 ± 25.5 *	52.5 ± 20.9 *	51.1 ± 4.12 *	41.6 ± 7.4
Stage 1 HTN	130–139	or 80–89	157.5 ± 25.2 *^#^	130.2 ± 20.6 *^#^	57.6 ± 4.08 *^#^	40.5 ± 9.2
Stage 2 HTN	≥140	or ≥90	194.6 ± 46.2 *^#†^	160.3 ± 37.8 *^#†^	68.9 ± 7.48 *^#†^	50.9 ± 6.6
**ESC/ESH 2018 HTN Guideline [14]**						
**(Original Population, *n* = 3022)**						
BP Category (mmHg)	SBP	DBP	TAC Score	TAC Volume (mm^3^)	TAC Density (HU)	CAC Score
Normal (NBP)	<130	and <85	31.7 ± 16.8	29.4 ± 13.7	29.2 ± 2.71	39.7 ± 4.2
High Normal (HNBP)	130–139	and/or 85–89	41.2 ± 23.0 *	35.0 ± 18.9 *	39.4 ± 3.71 *	55.6 ± 8.2
Grade 1 HTN	140–159	and/or 90–99	38.0 ± 28.5 *	32.0 ± 23.3 *	47.9 ± 4.60 *^#^	40.7 ± 8.1
Grade 2 HTN	≥160	and/or ≥100	152.2 ± 20.5 *^#†^	127.6 ± 16.8 *^#†^	64.5 ± 3.31 *^#†^	62.7 ± 15.1

Data presented as mean ± SD. * *p* < 0.01 compared to NBP, ^#^ *p* < 0.01 compared to EBP or HNBP, ^†^ *p* < 0.01 compared to stage 1 or grade 1 HTN. Model adjusted for age, sex, BMI, heart rate, HDL, LDL, eGFR, hyperlipidemia, CVD, diabetes, and smoking.

**Table 3:** Probability of the presence of TAC and CAC, according to the backward, stepwise, uni- and multi-variate regression models.

Logistic Regression (Total *n* = 3022)	Uni-Variate		Multi-Variate	
ACC/AHA 2017 HTN Guideline [2]	Odds Ratio		Odds Ratio	
Thoracic aortic calcium	OR (95% CI)	*p*-Value	OR (95% CI)	*p*-Value
Age (>50 years)	8.62 (6.64–11.20)	<0.001	7.01 (5.31–9.24)	<0.001
Sex (male)	0.86 (0.69–1.06)	0.15	—	NS
BMI (>25 kg/m^2^)	1.22 (1.01–1.48)	0.042	—	NS
Body fat (M > 23%, F > 25%) [12]	1.22 (0.99–1.50)	NS	—	NS
eGFR (<60 mL/min/1.73^2^)	4.18 (2.84–6.15)	<0.001	2.15 (1.39–3.33)	0.001
HTN (Stages 1 + 2)	3.60 (2.95–4.41)	<0.001	2.49 1.98–3.12)	<0.001
Diabetes	3.93 (2.86–5.40)	<0.001	1.83 (1.28–2.63)	0.001
CVD	3.33 (2.31–4.79)	<0.001	—	NS
Dyslipidemia	1.94 (1.35–2.79)	<0.001	—	NS
Smoking	1.20 (1.05–1.37)	0.008	—	NS
Coronary artery calcium				
Age (>50 y/o)	4.22 (3.57–4.99)	<0.001	4.17 (3.46–5.03)	<0.001
Sex (male)	2.37 (1.95–2.87)	<0.001	2.58 (2.08–3.21)	<0.001
BMI (>25 kg/m^2^)	1.95 (1.67–2.28)	<0.001	1.61 (1.34–1.93)	<0.001
Body fat (M > 23%, F > 25%)	1.18 (1.00–1.40)	0.048	—	NS
eGFR (<60 mL/min/1.73^2^)	1.99 (1.36–2.92)	<0.001	—	NS
HTN (Stages 1 + 2)	2.98 (2.54–3.49)	<0.001	2.03 (1.69–2.44)	<0.001
Diabetes	3.33 (2.43–4.55)	<0.001	1.74 (1.22–2.48)	0.002
CVD	2.54 (1.79–3.61)	<0.001	—	NS
Dyslipidemia	1.60 (1.16–2.22)	0.004	—	NS
Smoking	1.12 (0.88–1.41)	0.34	—	NS

BMI: body mass index, WHR: waist-to-hip ratio, HTN (1 + 2): stage 1 plus stage 2 hypertension, NS: not significant. Other abbreviations are the same as Table 1.

## Data Availability

The data presented in this study are available on request from the corresponding author. The data are not publicly available since these are admittedly completely anonymized patient data in clinical context.

## Supplementary Materials

The following are available online at https://www.mdpi.com/article/10.3390/diagnostics11061027/s1, Table S1: Sensitivity analysis exploring adjusted estimates of TAC burden across HTN stages/grades using different clinical guidelines in subjects without known CVD or onggoing HTN medication use. Table S2: Sensitivity analysis exploring the probability of TAC and CAC existence by backward stepwise uni- and multi-variate regression models in subjects without known CVD or onggoing HTN medication use

## Abbreviations

BP	blood pressure
CT	computed tomography
CAC	coronary artery calcification
CVD	cardiovascular diseases
DBP	diastolic blood pressure
HF	heart failure
HFpEF	heart failure with preserved ejection fraction
HTN	hypertension
MDCT	multi-detector computed tomography
PP	pulse pressure
SBP	systolic blood pressure
TAC	systolic blood pressure
