# Supplementary material for: Thoracic Aortic Calcification and Pre-Clinical Hypertension by New 2017 ACC/AHA Hypertension Guidelines
Source: Diagnostics (Basel). 2021 Jun 3;11(6):1027. doi: 10.3390/diagnostics11061027 (PMC8226485; doi:10.3390/diagnostics11061027)
Supplement: Supplementary file 1 [file diagnostics-11-01027-s001.zip › diagnostics-1178516-supplementary.pdf]

(Supplemental Materials)

Supplemental Table S1. Sensitivity analysis exploring the probability of TAC and CAC existence by backward stepwise uni- and multi-variate regression models in subjects without known CVD or onggoing HTN medication use

**Logistic regression *(Total n = 2,784)***	**Uni-variate**		**Multi-variate**	
**ACC/AHA 2017 HTN Guideline[2]**	**Odds ratio**		**Odds ratio**	
**Thoracic Aortic Calcium**	OR (95% CI)	P value	OR (95% CI)	P value
Age (>50 years)	7.98 (6.03-10.56)	<0.001	7.24 (5.35-9.79)	<0.001
Sex (Male)	0.86 (0.69-1.06)	0.15	—	NS
BMI (>25 kg/m^2)	1.24 (1.00-1.55)	0.05	—	NS
Body Fat (M>23%, F>25%)[12]	1.19 (0.94-1.50)	NS	—	NS
HTN (Stage 1+2)	3.24 (2.60-4.04)	<0.001	2.51 (1.94-3.24)	<0.001
Diabetes	3.49 (2.31-5.27)	<0.001	1.93 (1.19-3.14)	0.001
Dyslipidemia	1.40 (0.79-2.48)	0.25	—	NS
Smoking	1.12 (0.96-1.31)	0.15	—	NS
**Coronary Artery Calcium**				
Age (>50 y/o)	3.96 (3.31-4.74)	<0.001	4.15 (3.39-5.08)	<0.001
Sex (Male)	2.48 (2.00-3.07)	<0.001	2.76 (2.02-3.77)	<0.001
BMI (>25 kg/m^2)	1.92 (1.62-2.27)	<0.001	1.55 (1.21-1.99)	0.001
Body Fat (M>23%, F>25%)	1.10 (0.92-1.32)	0.31	—	NS
HTN (Stage 1+2)	2.66 (2.24-3.17)	<0.001	1.95 (1.60-2.39)	<0.001
Diabetes	2.80 (1.89-4.13)	<0.001	1.65 (1.05-2.61)	0.031
Dyslipidemia	1.00 (0.61-1.63)	0.99	—	NS
Smoking	1.20 (1.06-1.36)	0.003	—	NS

Supplemental Table S2. Sensitivity analysis exploring adjusted estimates of TAC burden across HTN stages/grades using different clinical guidelines in subjects without known CVD or onggoing HTN medication use

**ACC/AHA 2017 HTN Guideline[2]**					
(Sensitivity Analysis, n=2,784)					
BP Category (mmHg)	SBP	DBP	TAC Score	TAC Volume (mm^3)	TAC Density (HU)
Normal (NBP)	< 120	and <80	28.0 ±12.8	26.0 ±10.5	24.5 ±2.56
Elevated (EBP)	120-129	and <80	37.0 ±17.7[*]	31.3 ±14.4[*]	35.1 ±3.53[*]
Stage 1 HTN	130-139	or 80-89	32.4 ±22.1	27.2 ±18.0	45.1 ±4.41[*#]
Stage 2 HTN	≥ 140	or ≥ 90	138.9 ±18.8[*#†]	114.8 ±15.6[*#†]	59.7 ±3.76[*#†]
**ESC/ESH 2013 HTN Guideline[13]**					
(Sensitivity Analysis, n=2,784)					
BP Category (mmHg)	SBP	DBP	TAC Score	TAC Volume (mm^3)	TAC Density (HU)
Normal (NBP)	< 130	and < 85	32.6 ±10.7	29.0 ±8.73	29.8 ±2.15
High Normal (HNBP)	130-139	and/or 85-89	38.4 ±21.0[*]	32.2 ±17.1[*]	43.2 ±4.20[*]
Grade 1 HTN	140-159	and/or 90-99	151.1 ±21.4[*#]	124.6 ±17.5[*#]	51.7 ±4.30[*#]
Grade 2 HTN*	≥ 160	and/or ≥ 100	81.1 ±39.4[*#†]	68.9 ±32.1[*#†]	65.0 ±7.90[*#†]
